# Drug-induced torsades de pointes: Disproportionality analysis of the United States Food and Drug Administration adverse event reporting system

**DOI:** 10.3389/fcvm.2022.966331

**Published:** 2022-10-24

**Authors:** Ziyang Wu, Pengxiang Zhou, Na He, Suodi Zhai

**Affiliations:** ^1^Department of Pharmacy, Peking University Third Hospital, Beijing, China; ^2^Institute for Drug Evaluation, Peking University Health Science Center, Beijing, China

**Keywords:** long QT syndrome, torsades de pointes, FAERS, disproportionality analysis, pharmacovigilance

## Abstract

**Objective:**

This study aimed to identify the most common and top drugs associated with the risk of torsades de pointes (TdP) based on the United States Food and Drug Administration (FDA) Adverse Event Reporting System (FAERS) database.

**Materials and methods:**

We used OpenVigil 2.1 to query FAERS database and data from the first quarter of 2004 to the third quarter of 2021 were retrieved. The Medical Dictionary for Regulatory Activities (MedDRA) was used to identify TdP cases. We listed the most common drugs associated with the reported TdP cases. Then, the reporting odds ratio (ROR) and the proportional reporting ratio (PRR) for the reporting association between different drugs and TdP risk were calculated. Meanwhile, comparisons were conducted with the QT drug lists of CredibleMeds^®^ in an attempt to identify drugs with a potential risk of TdP that were not on the list.

**Results:**

A total of 9,217,181 adverse event reports were identified, of which 3,807 (0.04%) were related to TdP. TdP was more likely to occur in the elderly and females. Amiodarone (464 cases) was associated with most cases of TdP. According to the disproportionality analysis, the top five drugs with the highest ROR and PRR were tolazoline (ROR 1615.11, 95% confidence interval [CI] 455.59–5725.75, PRR 969.46, χ^2^ 2960.10), levomethadyl (ROR 1211.01, 95% CI 302.75–4844.04, PRR 807.67, χ^2^ 1677.03), ibutilide (ROR 1118.74, 95% CI 425.00–2944.91, PRR 765.77, χ^2^ 3845.27), halofantrine (ROR 660.55, 95% CI 184.21–2368.69, PRR 519.22, χ^2^ 1076.31), and isoproterenol (ROR 352.20, 95% CI 227.19–546.00, PRR 307.82, χ^2^ 6692.53). Approximately half of the top 50 drugs (22 for ROR, 30 for PRR) were not outlined on the QT drug lists of CredibleMeds^®^.

**Conclusion:**

Approximately half of the top risk drugs (22 for ROR, 30 for PRR) were not outlined in the QT drug lists of CredibleMeds^®^. Notably, potential risks are of great importance and should be closely monitored in clinical practice. Also, further research is needed to investigate the association between these drugs and TdP.

## Introduction

Torsades de pointes (TdP) is a type of polymorphic ventricular tachycardia (1). The incidence of TdP is from 0.0032‰/year to 0.16‰/year (2–4), which is very low but often life-threatening. The mortality of TdP is approximately 10–20% (5). In general, TdP is associated with prolonged QT interval, for every 10 ms increase in QT interval, the risk of TdP increases by approximately 5–7% (6). Long QT interval can be congenital or acquired. The latter is most often drug-induced. In clinical practice, multiple drugs may cause TdP (7, 8), and several of them have been withdrawn from the market due to TdP, such as cisapride, droperidol, and terfenadine (9, 10).

It is particularly essential to assess drug-induced TdP. The International Conference on Harmonization of Technical Requirements for Registration of Pharmaceuticals for Human Use (ICH) released a guideline for clinical evaluation of QT/QTc interval prolongation. This guideline could help us identify drugs that may cause QT prolongation before marketing (11). However, due to the limited sample size and follow-up time, it is difficult to observe rare adverse events (AEs) in such studies. Therefore, post-marketing surveillance is also very important, especially for rare but clinically significant AEs, such as TdP. Food and Drug Administration Adverse Event Reporting System (FAERS) database is believed to be a powerful tool for mining new or rare AEs and could reflect profiles of AEs in real-world clinical settings. It is a publicly accessible database that includes a large number of AE reports submitted by healthcare professionals, consumers, and manufacturers (12). Previous researchers have used the FAERS database to investigate TdP and have comprehensively assessed the specific class drugs in terms of TdP, mainly including H_1_-antihistamines, antipsychotics, and antibiotics (13–16).

Increasing new drugs have been approved on the market in recent years, and it is of great significance to update the risk of TdP in currently available drugs based on spontaneous reporting AEs data. Therefore, this study aimed to comprehensively investigate the risk of drug-induced TdP across all drugs and identify drugs with a potential risk of TdP that were not on the QT drug lists of CredibleMeds^®^.

## Materials and methods

### Data source

This retrospective pharmacovigilance study was conducted based on the FAERS database. FAERS is a spontaneous reporting AEs database which is available to the public. A large amount of information of AEs can be found in this database, such as demographic and administrative information, drug information and reaction information (17, 18).

### Data collection

OpenVigil 2.1^1^ was used to retrieve FAERS data. OpenVigil 2.1 is a validated pharmacovigilance data extraction, cleaning, and mining tool of the FAERS database (19, 20). For this study, the AEs of TdP were searched from the first quarter of 2004 to the third quarter of 2021 using preferred terms (PTs) in the Medical Dictionary for Regulatory Activities (MedDRA) Dictionary (Version 24.0). We used Torsade de Pointes (PT: 10044066) to search.

### Statistical analysis

First, the descriptive analysis was performed to summarize the clinical features of cases of TdP, including patients’ gender, age and reporting country. According to the counts of reports, the top 50 drugs associated with TdP were selected.

Second, a disproportionality analysis was conducted. Disproportionality analysis is largely used to generate hypotheses on possible associations between drugs and AEs. It is based on the contrast between observed and expected numbers of reports, for any given drug and AE (21). Reporting odds ratio (ROR) and proportional reporting ratio (PRR) were two measures of disproportionality analysis. We calculated ROR and PRR to detect each drug’s TdP risk signal. The equations and criteria for the algorithms were listed in Table 1 (22–24). ROR and PRR offer a rough indication of the strength of the signal. A relatively higher ROR or PRR indicates a stronger signal between the drug and TdP. The analyses were performed using the Microsoft EXCEL 2019.

**TABLE 1:** Summary of algorithms used for signal detection.

Algorithms	Equation	Criteria
ROR	ROR = ad/bc	lower limit of 95% CI > 1, a ≥ 2
	95%CI = e^ln(ROR)±1^.^96(1/a+1/b+1/c+1/d)∧0^.^5^	
PRR	PRR = a(c + d)/c(a + b)	PRR ≥ 2, χ^2^ ≥ 4, a ≥ 3
	χ^2^ = [(ad − bc)^2^](a + b + c + d)/[(a + b)(c + d)(a + c)(b + d)]	

a, number of reports containing both the suspect drug and the suspect adverse drug reaction; b, number of reports containing the suspect adverse drug reaction with other medications (except the drug of interest); c, number of reports containing the suspect drug with other adverse drug reactions (except the event of interest); d, number of reports containing other medications and other adverse drug reactions. ROR, reporting odds ratio; PRR, proportional reporting ratio; χ^2^, chi-squared.

Credible Meds (accessible at www.crediblemeds.org) has established a list of drugs that increase risk of QT prolongation and TdP. And these drugs were classified into three categories, “known risk,” “possible risk,” or “conditional risk” of TdP. In our study, comparisons were conducted with the QT drug lists of CredibleMeds^®^ in an attempt to identify drugs with a potential risk of TdP that are not on the list (25).

## Results

### Descriptive analysis

From 2004 Q1 to 2021 Q3, there were 9,217,181 AEs in FAERS, of which 3,807 (0.04%) were TdP cases. The characteristics of cases were listed in Table 2. TdP was more likely observed in female patients (56.82%) and most frequently reported in the United States (41.27%). When stratified by age group, the majority of AE reports were distributed to the elderly (≥65 years) (33.44%).

**TABLE 2:** Characteristics of cases with torsades de pointes.

Characteristics	Cases, *n* (%) (Total cases: 3807)
**Age**	
≤18	154 (4.04)
19–40	646 (16.97)
41–64	1093 (28.71)
≥65	1273 (33.44)
Unknown or missing	641 (16.84)
**Gender**	
Male	1306 (34.31)
Female	2163 (56.82)
Unknown or missing	338 (8.87)
**Reporting country**	
United States	1571 (41.27)
United Kingdom	286 (7.51)
Canada	186 (4.89)
Japan	175 (4.60)
Germany	154 (4.05)
China	29 (0.75)
Other regions	1406 (36.93)

**FIGURE 1:**
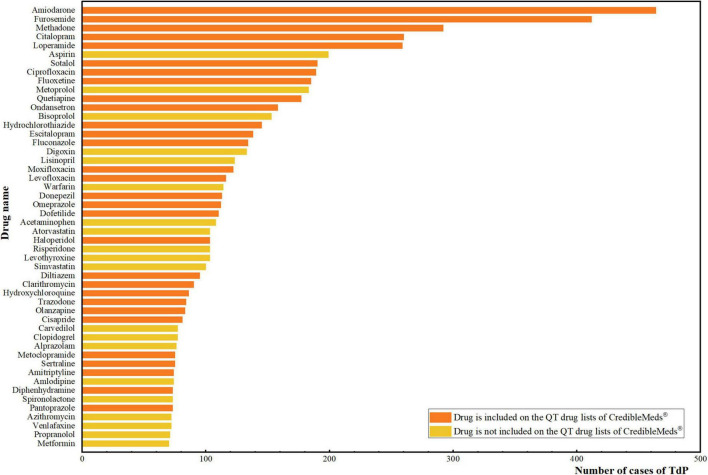
Top 50 drugs with the most number of cases of TdP. The vertical axis is the name of the drugs, and the horizontal axis is the corresponding number of cases of TdP for each drug. The orange color represents that the drug is included on the QT drug lists of CredibleMeds^®^. The yellow color represents that the drug is not included on the QT drug lists of CredibleMeds^®^.

Based on the counts of AE reports, the 50 most common drugs associated with TdP are summarized in Figure 1. Among the most frequently reported drugs, amiodarone (464 cases) was associated with the most cases of TdP, followed by furosemide (412 cases), methadone (292 cases), citalopram (260 cases) and loperamide (259 cases). Of these 50 drugs, 30 were included on the QT drug lists of CredibleMeds^®^.

### Disproportionality analysis

Based on the criteria for ROR, a total of 306 signals were detected for TdP. Drugs with the top 50 highest RORs are listed in Table 3. Tolazoline (ROR 1615.11, 95%CI 455.59–5725.75) reported the highest ROR for TdP, followed by levomethadyl (ROR 1211.01, 95% CI 302.75–4844.04), ibutilide (ROR 1118.74, 95% CI 425.00–2944.91), halofantrine (ROR 660.55, 95% CI 184.21–2368.69) and isoproterenol (ROR 352.20, 95% CI 227.19–546.00). Of the top 50 drugs, 28 were included on the QT drug lists of CredibleMeds^®^. According to the risk categories of CredibleMeds^®^, 19 drugs were classified as known risk of TdP, 5 drugs as conditional risk, 3 drugs as special risk, and the other one drug as possible risk of TdP.

**TABLE 3:** Reported odds ratios for the top 50 drugs.

Drug name	ROR (95% CI)	CredibleMeds^®^ TdP risk
Tolazoline	1615.11 (455.59–5725.75)	N
Levomethadyl	1211.01 (302.75–4844.04)	KR
Ibutilide	1118.74 (425.00–2944.91)	KR
Halofantrine	660.55 (184.21–2368.69)	KR
Isoproterenol	352.20 (227.19–546.00)	SR
Chlorcyclizine	302.67 (69.57–1316.85)	N
Cisapride	273.60 (217.01–344.95)	KR
Viloxazine	134.52 (32.38–558.91)	N
Thiamylal	103.80 (32.67–329.81)	N
Procainamide	82.63 (36.60–186.55)	KR
Bepridil	78.68 (32.27–191.82)	KR
Safflower oil	78.11 (19.10–319.44)	N
Tandospirone	72.28 (17.70–295.12)	N
Sotalol	70.10 (60.47–81.26)	KR
Sertindole	62.09 (15.25–252.77)	KR
Amsacrine	61.33 (22.73—165.53)	CR
Esmolol	60.07 (29.76–121.22)	N
Amrinone	56.31 (13.85–228.88)	N
Droperidol	53.35 (30.09–94.62)	KR
Clemastine	52.90 (32.20–86.90)	N
Vorinostat	48.82 (32.27–73.85)	PR
Dofetilide	47.71 (39.40–57.78)	KR
Ferrous sulfate anhydrous	47.05 (24.31–91.07)	N
Disopyramide	46.90 (27.61–79.67)	KR
Amiodarone	46.88 (42.51–51.69)	KR
Fluphenazine	46.19 (31.94–66.78)	N
Ticarcillin	43.77 (13.97–137.17)	N
Almotriptan	38.68 (17.26–86.70)	N
Mexiletine	37.65 (20.74–68.36)	N
Terfenadine	37.25 (9.22–150.60)	KR
Ivabradine	35.39 (26.41–47.43)	CR
Pancuronium	34.24 (12.76–91.91)	N
Dopamine	32.59 (20.70–51.30)	SR
Pimozide	31.39 (12.98–75.89)	KR
Methadone	29.08 (25.79–32.79)	KR
Loperamide	29.03 (25.57–32.96)	CR
Sevoflurane	27.28 (19.61—37.95)	KR
Flecainide	26.84 (21.01–34.28)	KR
Acamprosate	26.53 (13.74–51.20)	N
Acenocoumarol	25.55 (18.61–35.08)	N
Flucytosine	25.50 (9.52–68.33)	N
Betahistine	24.04 (14.89–38.80)	N
Fluindione	23.85 (15.33–37.09)	N
Cimetidine	23.40 (16.49–33.19)	CR
Dronedarone	22.81 (17.28–30.11)	KR
Isoflurane	22.81 (10.83–48.05)	N
Chloral hydrate	22.09 (7.08—68.86)	CR
Ritodrine	21.06 (5.23–84.73)	SR
Cloxacillin	20.88 (6.70–65.08)	N
Chloroquine	20.51 (11.87–35.44)	KR

ROR, reporting odds ratio; CI, confidence interval; KR, known risk; PR, possible risk; CR, conditional risk; SR, special risk; N, not on the list.

The estimated PRRs for each of the individual drugs associated with TdP are summarized in the Supplementary Table 1. A total of 253 signals were detected, and the top five drugs with the highest PRRs were consistent with the results of RORs, including tolazoline (PRR 969.46, χ^2^ 2960.10), levomethadyl (PRR 807.67, χ^2^ 1677.03), ibutilide (PRR 765.77, χ^2^ 3845.27), halofantrine (PRR 519.22, χ^2^ 1076.31), and isoproterenol (PRR 307.82, χ^2^ 6692.53). Thirty drugs were included on the QT drug lists of CredibleMeds^®^. According to the risk categories, 22 drugs were classified as known risk of TdP, 5 drugs as conditional risk, 2 drugs as special risk, and the other one drug as possible risk of TdP.

## Discussion

This study comprehensively assessed the AEs of drug-induced TdP in the real world based on the FAERS database. The results indicated that TdP was more likely to occur in the elderly and females. Amiodarone was associated with most cases of TdP. According to the disproportionality analysis, the top five drugs with the highest ROR and PRR were tolazoline, levomethadyl, ibutilide, halofantrine and isoproterenol. Approximately half of the top 50 drugs (22 for ROR, 30 for PRR) were not outlined on the QT drug lists of CredibleMeds^®^.

Drug-induced TdP is a non-negligible AE in pharmaceutical treatments, which can lead to cardiac sudden death. Previous studies have found multiple risk factors for TdP, including female, age (≥65 years), etc. (26–28), which was also observed in our study. Sex-related differences in TdP are increasingly recognized, but the mechanisms remain uncertain. Sex hormones may be a possible factor for sex-related differences. Testosterone appears to shorten the QT interval, and estrogen may lengthen QT interval. Thus, females have a longer QT interval compared to males after puberty, and the QT interval in males gradually lengthens and approximates that of females with aging (29, 30). In addition, QTc > 500 ms is considered a risk factor of TdP. Females have a higher baseline QT interval and are more likely to prolong over 500 ms when challenged by QT-prolonging drugs (7). In terms of the elderly, their prescriptions have more QT-prolonging drugs. A study conducted on 5,319 elderly outpatients in North Jordan showed that 58.5% of patients were consuming drugs that carry the risk of TdP (31). Therefore, the elderly are more likely to be exposed to high risk of TdP.

Based on data mining, we listed the top 50 drugs with the strongest signals, and compared these drugs to the QT drug lists of CredibleMeds^®^. Interestingly, about half of the drugs (22 for ROR, 30 for PRR) were not outlined in lists, representing potential new signals. The drugs involved in new signals include antihistamines (e.g., clemastine), antibiotics (e.g., cloxacillin), inhalation anesthetics (e.g., isoflurane), etc. Although some of these drugs have been on the market for a long time, there is inadequate attention to TdP. Therefore, further attention is needed to determine whether these drugs are needed to be included on the QT drug lists of CredibleMeds^®^. On the other hand, the post-marketing safety monitoring of newly marketed drugs is also an imperative topic. In our study, of the top 50 drugs, viloxazine was the most recently marketed (32). Due to the short time on the market, only two cases of TdP were reported, but with significantly higher ROR and PRR. As a result, physicians and pharmacists should be alert for viloxazine-induced TdP in clinical practice.

CredibleMeds is one of the most reliable sources of information on drug-induced QT prolongation, with close monitoring of the FAERS database as well. Our results are slightly different from the QT drug lists of CredibleMeds^®^ may be due to different methods. The CredibleMeds team used the Bayesian method to estimate the relative reporting ratio and we used the frequentist method. These two methods have their own strengths. The frequentist method has higher sensitivity, while the Bayesian method has higher specificity (33). Therefore, we hope our results could be a supplement to detect TdP of drugs.

Our study has several strengths. Above all, FARES is one of the largest public pharmacovigilance databases, and the sample size is sufficient to detect rare AEs of TdP and provide valuable suggestions to guide clinical decision-making. Further, we compared the 50 drugs with the strongest signals to the QT drug lists of CredibleMeds^®^ and mined some new signals, which can be considered hypotheses to stimulate further research. Last but not least, given their simplicity and sensitivity, the choice of ROR and PRR for signal detection helps to detect new AEs signals more quickly.

Our study also has several limitations. First of all, due to the missing denominator data, the true incidence of TdP of each drug was not estimated based on the FAERS database (34). Second, considering that FAERS is a spontaneous reporting system, the causal relationship between AEs and drugs may not be determined. Thus, our research can only give clinicians and researchers some hints about which drugs may have a high potential risk of TdP, and further studies with more reliable methods are needed to verify. Third, due to the insufficient information in the FAERS database, it is hard to assess the effects of drug-drug interactions or route of administration (e.g., oral vs. intravenous) on TdP. Finally, underreporting may not be avoided because prolongation of the QT intervals is judged by an electrocardiogram, which is not routinely equipped in clinical practice. Nevertheless, the FAERS database remains an important tool for post-marketing surveillance.

## Conclusion

In conclusion, we comprehensively assessed TdP reports and associated drugs using the FAERS database. Approximately half of the top risk drugs (22 for ROR, 30 for PRR) were not outlined on the QT drug lists of CredibleMeds^®^. Notably, the potential risks are of great importance and should be closely monitored in clinical practice. Also, further research is needed to investigate the association between these drugs and TdP.

## Data availability statement

Publicly available datasets were analyzed in this study. This data can be found here: http://h2876314.stratoserver.net:8080/OV2/search/.

## Author contributions

ZW and PZ extracted and analyzed the data and drafted the manuscript. SZ provided pharmacological guidance. NH supported data analysis. All authors participated in the study design, contributed to the revision of the manuscript, and approved the final version.

## Footnotes

1
http://h2876314.stratoserver.net:8080/OV2/search/
