# Supplementary material for: Drug-induced torsades de pointes: Disproportionality analysis of the United States Food and Drug Administration adverse event reporting system
Source: Front Cardiovasc Med. 2022 Oct 24;9:966331. doi: 10.3389/fcvm.2022.966331 (PMC9639787; doi:10.3389/fcvm.2022.966331)
Supplement: Supplementary file 1 [file Table_1.DOCX]

Supplementary Material

**Supplementary Table 1 Proportional reporting ratio for the top 50 drugs**

| **Drug name** | **PRR (χ^2^)** | **CredibleMeds**^®^ **TdP risk** |
| --- | --- | --- |
| tolazoline | 969.46 (2960.1) | **N** |
| levomethadyl | 807.67 (1677.03) | KR |
| ibutilide | 765.77 (3845.27) | KR |
| halofantrine | 519.22 (1076.31) | KR |
| isoproterenol | 307.82 (6692.53) | SR |
| cisapride | 246.44 (19147.12) | KR |
| thiamylal | 99.58 (202.4) | **N** |
| procainamide | 79.94 (391.66) | KR |
| bepridil | 76.23 (299.54) | KR |
| sotalol | 68.25 (11902.46) | KR |
| amsacrine | 59.84 (176.22) | CR |
| esmolol | 58.64 (396.76) | **N** |
| droperidol | 52.23 (551.31) | KR |
| clemastine | 51.8 (744.21) | **N** |
| vorinostat | 47.88 (1003.62) | PR |
| dofetilide | 46.83 (4749.05) | KR |
| ferrous sulfate anhydrous | 46.18 (353.18) | **N** |
| amiodarone | 46.11 (17947.5) | KR |
| disopyramide | 46.03 (570.68) | KR |
| fluphenazine | 45.35 (1204.96) | **N** |
| ticarcillin | 43.01 (84.65) | **N** |
| almotriptan | 38.09 (180.96) | **N** |
| mexiletine | 37.09 (350.2) | **N** |
| ivabradine | 34.9 (1463.73) | CR |
| pancuronium | 33.78 (96.5) | **N** |
| dopamine | 32.17 (540.54) | SR |
| pimozide | 31 (116.61) | KR |
| methadone | 28.77 (7204.73) | KR |
| loperamide | 28.72 (6434.65) | CR |
| sevoflurane | 26.99 (867.25) | KR |
| flecainide | 26.56 (1570.75) | KR |
| acamprosate | 26.25 (193.69) | **N** |
| acenocoumarol | 25.3 (877.19) | **N** |
| flucytosine | 25.25 (70.43) | **N** |
| betahistine | 23.81 (347.62) | **N** |
| fluindione | 23.63 (409.02) | **N** |
| cimetidine | 23.18 (651.97) | CR |
| dronedarone | 22.61 (1018.45) | KR |
| isoflurane | 22.61 (123.58) | **N** |
| chloral hydrate | 21.89 (40.73) | CR |
| cloxacillin | 20.71 (38.27) | **N** |
| chloroquine | 20.35 (219.52) | KR |
| donepezil | 20.04 (1966.17) | KR |
| thiopental | 19.31 (103.79) | **N** |
| indinavir | 19.19 (137.26) | **N** |
| cefixime | 18.55 (115.69) | **N** |
| sulpiride | 18.07 (176.28) | KR |
| fluconazole | 17.74 (2027.17) | KR |
| domperidone | 17.65 (544.02) | KR |
| fluconazole | 17.74 (2027.17) | KR |
